# Circulating MicroRNA-21 Is a Potential Diagnostic Biomarker in Gastric Cancer

**DOI:** 10.1155/2015/435656

**Published:** 2015-05-03

**Authors:** Jianhong Wu, Guangxin Li, Zeyou Wang, Yongliang Yao, Rui Chen, XiongYong Pu, Jianjun Wang

**Affiliations:** ^1^Department of Clinical Laboratory, Kunshan First People's Hospital, Jiangsu University, Kunshan 215300, China; ^2^Department of Pathology, Chongqing Cancer Institute, Chongqing 400030, China; ^3^Institute of Cancer Research, Central South University, Changsha 410078, China

## Abstract

MicroRNA-21 was upexpressed in gastric cancer (GC) indicating that it is a potential diagnostic biomarker for GC. In this study, 50 GC patients and 50 healthy controls were recruited. miR-21 levels in serum and peripheral blood mononuclear cells (PBMCs) were quantified using quantitative real-time PCR. CA199, and CEA were measured using electrochemiluminescence assay. The sensitivity and specificity of circulating miR-21, CA199 and CEA in GC diagnosis, the correlation of circulating miR-21 to clinicopathological features, and the diagnostic value of miR-21 in different GC stages were determined. The levels of miR-21 in both serum and PBMCs increased significantly in GC patients comparing to healthy controls; however, no correlation was observed between circulating miR-21 level and clinicopathological features. The sensitivity and specificity of miR-21 in serum and PBMCs, and CA199 and CEA in GC diagnosis were 88.4%, 79.6%, 81.3%, 73.4%, 60.5%, 55.9%, and 68.6%, 59.3%, respectively. The positive prediction rates of circulating miR-21 in GC stages I to IV were all around 90%, while those of CA199 and CEA were around or less than 50%. Our data suggest circulating miR-21 (both in serum and in PBMCs) can serve as a good biomarker for GC and could be used in diagnosis of early (stage I) and late GC (stage IV).

## 1. Introduction

MicroRNAs (miRNAs), small single-strand RNA molecules with 18–25 nucleotides in length, possess the ability to modulate gene expression at posttranscription level [1, 2]. Extensive research has revealed that miRNAs are involved in multiple biological processes including cell proliferation, differentiation, and apoptosis as well as development [3]. Among these identified miRNAs, many of them have demonstrated modulation in initiation and progression of various types of cancers [4–7].

miRNA-21 (miR-21), one of the first identified and most prevalent miRNAs in human cells, has been studied in various diseases including cardiovascular diseases as well as cancers. Particularly, since the miR-21-targeted genes identified till now are mostly tumor suppressors, miR-21 is closely related to various types of cancers including hepatocellular cancer [8], glioblastoma [9], glioma [10], and laryngeal carcinoma [11] and has been designated as an oncomir [12–14]. Clinical research has revealed that the expression of miR-21 is elevated in a wide range of cancers including brain, breast, cervix, lung, liver, prostate, pancreas, and colon [15–22]. Due to the association with cancers, the potential of miR-21 as a cancer biomarker has also been widely studied for the past few years. In colorectal cancer, serum miR-21 could serve as a promising indicator for early detection as well as prognosis [23]. In colon adenocarcinoma, high level of miR-21 indicates poor therapeutic outcome and survival [24], whereas in lung cancer, serum miR-21 is diagnostic indicator with moderate sensitivity and specificity [25].

Gastric cancer (GC) is the second most common cancer around the world and is responsible for almost one million deaths per year worldwide. The high death rate is partially due to the lack of effective means for GC early screening. Cancer antigen 199 (CA199) and carcinoembryonic antigen (CEA) are two common tumor diagnostic markers; however, their specificity and sensitivity are too low for GC diagnosis. Therefore, a good biomarker of screening for GC is urgently needed. Previous studies have revealed that miR-21 has implications in GC progression. In vitro and ex vivo studies have shown that this microRNA is expressed in aberrantly high level in gastric cancer cell lines as well as primary tissues [26]. Moreover, miR-21 is associated with differentiation of tumor tissues as well as survival rates [26]. The mechanism study even discovered that miR-21 promotes GC proliferation and invasion probably by targeting PTEN [27]. However, the potential value of miR-21 as a screening biomarker in GC has not yet been investigated.

In the current study, by recruiting 50 GC patients and 50 healthy controls, we systematically evaluated the potential of circulating (serum and peripheral blood mononuclear cells) miR-21 as a screening GC marker in comparison to conventional cancer markers CA199 and CEA.

## 2. Materials and Methods

### 2.1. Ethical Statement

All protocols involving human subjects in the study were reviewed and approved by the Ethical Committee of Jiangsu University in accordance with the Declaration of Helsinki [28]. Informed written consents were obtained from the subjects who participated in this study.

### 2.2. Sample Collection

Fifty GC patients and 50 healthy individuals were recruited at the Department of Clinical Laboratory, Kunshan First People's Hospital, Jiangsu University, in 2014 from February to October. GC patients were classified into four stages according to Borrmann's classification [29]. For each participant, a total volume of 10 mL peripheral blood was collected. Five millilitres was mixed with anticoagulant for peripheral blood mononuclear cell (PBMC) isolation, while the other 5 mL without anticoagulant for serum isolation. Blood samples with anticoagulant were stored on ice and sent for PBMC isolation in 1 h, while samples without anticoagulant were kept at room temperature and sent for serum isolation after coagulation.

### 2.3. Isolation of PBMCs and Serum Samples

PBMCs were isolated from whole blood samples using Ficoll-Paque Plus (GE healthcare) density gradient centrifugation according to the manufacturer's instructions. In brief, whole blood samples were layered on Ficoll-Paque Plus solution and centrifuged at 800 g for 30 min at 4°C. PBMC layer was then extracted and washed with PBS twice and centrifuged at 350 g for 10 min at 4°C. After washes, PBMCs were resuspended in PBS, aliquoted, and stored at −80°C till use. For serum isolation, coagulated blood samples were centrifuged at 1000 g for 10 min at 4°C and serum was collected and aliquoted and stored at −80°C till use.

### 2.4. Total RNA Extraction and miR-21 Quantification by Quantitative Real-Time PCR (qRT-PCR)

Total RNA was extracted from both serum and PBMCs using Trizol reagent (Invitrogen, Life Technologies) and the first strand cDNA was synthesized using PrimeScript RT Reagent Kit (Takara), both according to the manufacturer's instructions. miR-21 was quantified by qRT-PCR using U6 miRNA as control. The qRT-PCR was carried out using a SYBR Premix Ex Taq Kit (Takara) on a 7500 real-time PCR system (Applied Biosystems). The primers used for miR-21 amplification were (forward) 5′ACGTTGTGTAGCTTATCAGACTG3′ and (reverse) 5′AATGGTTGTTCTCCACACTCTC3′, and primers for U6 were (forward) 5′ATTGGAACGATACAGAGAAGATT3′ and (reverse) 5′GGAACGCTTCACGAATTTG3′. Each sample was determined in duplicate. The amplification specificity was validated by melting curve analysis and agarose gel electrophoreses of PCR products. miR-21 level was calculated relative to U6 miRNA using the 2^−ΔΔCt^ formula, where ΔΔCt = ΔCt_reference_ − ΔCt_sample_, ΔCt is the difference in the cycling threshold between miR-21 and U6, ΔCt_sample_ is the Ct value of U6-normalized miR-21, and ΔCt_reference_ is the Ct value corresponding to control samples normalized to U6.

### 2.5. CEA and CA199 Measurements

The levels of CEA and CA199 in serum were determined using electrochemiluminescence assay with a Roche E170 MODULAR Immunoassay Analyzer according to the manufacturer's instructions (Roche).

### 2.6. Statistical Analysis

Mann-Whitney test was adopted for the comparison of miR-21 expression difference between GC patients and healthy controls. Kruskal-Wallis test was used for the comparisons between miR-21 and CA199 and CEA. Spearman correlation test was used for correlation analysis. Receiver operating characteristic (ROC) curve was carried out for the diagnostic evaluation of circulating miR-21 in GC and the cut-off values were determined using a training dataset and then applied to the remaining population. All analyses were performed with SPSS 16.0 software (SPSS Inc.) and a *p* value < 0.05 was considered statistically significant.

## 3. Results

### 3.1. Both Serum and PBMC miR-21 Levels Were Significantly Elevated in GC Patients in comparison to Healthy Controls

**Figure 1:**
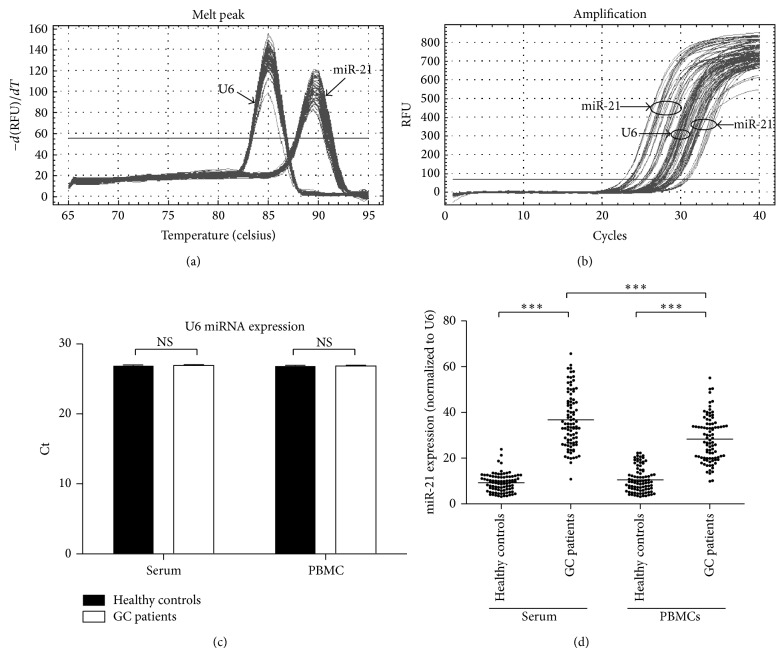
qRT-PCR quantification of miR-21 in serum and PBMCs of GC patients and healthy controls. (a) Amplification curves of miR-21 and U6. (b) Melting curves of miR-21 and U6. (c) Ct values of U6 in serum and PBMCS from GC patients and healthy controls. (d) miR-21 levels in serum and PBMCs of GC patients and healthy controls. NS: not statistically significant; ^∗∗∗^ *p* < 0.001.

Circulating miR-21 levels in both serum and PBMCs were quantified by qRT-PCR using U6 as normalization control. First, U6 and miR-21 amplification curves as well as melting curves were analysed to check the validation of the current qRT-PCR system. As shown in Figures 1(a) and 1(b), no nonspecific products were amplified in the reaction, indicating that the primers used in the current qRT-PCR system could specifically amplify miR-21 and U6, respectively. Further analysis of U6 levels in serum and PBMCs from GC patients and healthy controls revealed that the expression of this conserved miRNA remained at comparable levels in both serum and PBMCs among all participants in this study, which indicated that U6 was an appropriate normalization control for miR-21 quantification in serum and PBMCs (Figure 1(c)).

Following qRT-PCR validation, we subsequently measured the miR-21 levels in serum and PBMCs from GC patients and healthy controls. As shown in Figure 1(d), basal level of miR-21 could be detected in both serum and PBMCs from healthy controls, while the level of this microRNA was significantly increased in serum and PBMCs from GC patients (*p* < 0.001). Of note, the miR-21 elevation in serum was more profound than that in PBMCs (*p* < 0.001). Taken together, these results indicated that circulating (in serum and PBMCs) miR-21 elevation might be a concomitant clinical manifestation in GC disease.

### 3.2. miR-21 Level Was Not Associated with GC Clinicopathological Features

Subsequently, we further analysed the relationship of miR-21 with GC clinicopathological features. The analysis included clinical stage, age, gender, lymphatic metastasis, differentiation degree, and surgery history. Out of surprise, all the analysed variables, however, did not show any statistical correlation to miR-21 level in either serum or PBMCs (*p* > 0.05 for all determinations, Table 1).

### 3.3. Diagnostic Value of Circulating miR-21 in GC

**Figure 2:**
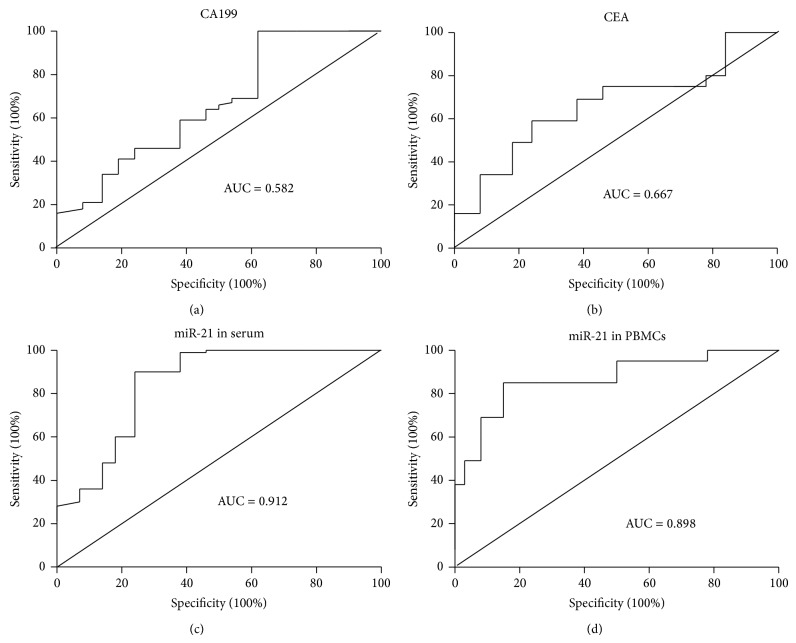
ROC analysis of CA199, CEA, and miR-21 in serum and PBMCs. An AUC value was given in each curve plot.

Since the elevation of miR-21 in serum and PBMCs was related to GC, we next determined whether circulating miR-21 elevation could serve as a diagnostic biomarker for this cancer. The diagnostic value of circulating miR-21 was evaluated in comparison to conventional tumor biomarkers CA199 and CEA. First, ROC curve analysis was conducted to determine the specificity and sensitivity of circulating miR-21 in GC diagnosis. As shown in Figure 2, the area under curve (AUC) value of CA199 was 0.582 (95% CI: 0.452–0.681), with the sensitivity of 60.5% and the specificity of 55.9% at the cut-off of 5.69. The AUC value of CEA was 0.667 (95% CI: 0.536–0.724), with the sensitivity of 68.6% and the specificity of 59.3% at the cut-off of 5.21. The AUC value of miR-21 in serum was 0.912 (95% CI: 0.869–0.968), with the sensitivity of 88.4% and the specificity of 79.6% at the cut-off of 2.78. The AUC value of miR-21 in PBMCs was 0.898 (95% CI: 0.838–0.935), with the sensitivity of 81.3% and the specificity of 73.4% at the cut-off of 3.02. These data indicated that circulating (both in serum and in PBMCs) miR-21, but not CA199 and CEA, had high odds in GC prediction. Further analysis by multivariate logistic regress confirmed that miR-21 in both serum (*p* < 0.01) and PBMCs (*p* < 0.05) was good GC biomarker while the two conventional tumor biomarkers CA199 and CEA were not good indicators for GC (*p* > 0.05 for both determinations). Of note, although serum miR-21 demonstrated slightly higher sensitivity and specificity than PBMC miR-21 in ROC curve analysis, the pairwise comparisons of the two ROC curves revealed that there was no statistical difference between miR-21 in serum and PBMCs in GC prediction (*p* > 0.05).

We next determined whether circulating miR-21 could be used as an indicator in the diagnosis of GC in different stages. Positive prediction rates of miR-21 in serum and PBMCs as well as conventional tumor biomarkers CA199 and CEA were calculated and compared based on the cut-off values from ROC curves (Figure 2 and Table 2). Our results showed that the positive rate of CA199 in prediction of GC stages I to IV was 44.44%, 54.54%, 40.00%, and 61.11%, respectively, with an overall positive rate of 50.00%. Similarly, the positive rate of CEA in prediction of GC stages I to IV was 33.33%, 45.45%, 50.00%, and 55.55%, respectively, with an overall positive rate of 46.00%. Unlike CA199 and CEA, the positive rates of miR-21 in serum and PBMCs were considerably higher (stages I to IV and overall GC positive rates of serum miR-21 were 88.88%, 90.90%, 90.00%, 94.44%, and 88.00% and of PBMCs miR-21 were 88.88%, 81.81%, 90.00%, 94.44%, and 84.00%). These data revealed that circulating miR-21 (both in serum and in PBMCs) was good biomarker for diagnosis of GC in all stages.

## 4. Discussion

With a total number of 700,000 GC-related deaths annually, GC has been the second most common cancer worldwide [30, 31]. However, after decades of research, there is still a lack of effective biomarkers for GC diagnosis. For most cancers, blood-based proteins have been proven and widely used as biomarkers in clinical diagnosis. Unfortunately, situation is quite different for GC. Common tumor biomarkers like CA125, CA199, and CEA have exhibited poor diagnostic value in GC [32]. Consequently, the discovery of GC diagnostic biomarkers is in critical need. miRNAs, a group of regulatory small molecular RNAs, have been reported to be correlated with the progression of various cancers and some of them even have potential as biomarkers in cancer diagnosis [33–35]. In the current study, our research discovered that circulating miR-21 (detected in serum and PBMCs) was significantly elevated in GC patients and could be a potential biomarker for GC diagnosis. Of note, our research also found that miR-21 as a biomarker was applicable for GC in all stages. Although it is beyond the scope of the current study, further research is warranted to investigate whether other miRNAs are also capable of indicating GC progression and more importantly whether miR-21 is the best choice among potential GC biomarkers.

Diagnostic tests through noninvasive means are preferable, peripheral blood-based tests and therefore are widely used in clinical diagnosis. Circulating miRNAs as novel tumor biomarkers have demonstrated promising results in preclinical research and have been given great hope as measures for future cancer diagnosis. However, the aberrant elevation of miRNAs is originated from cancer tissues while some studies have suggested that miRNA expression profile was not identical to that observed in cancer tissues [36, 37]. Therefore, to what extent could the circulating microRNA level reflect the variation in cancer tissues is still needed to be further investigated in each type of cancers. In our study, miR-21 in blood is originated from GC tissues in GC patients, so further research is needed to determine the correlation of miR-21 levels between blood-based samples (e.g., serum and PBMCs) and GC tissues or gastric juice.

In addition, blood is a complex sample containing a variety of cells as well as many other components in the plasma. It is highly possible that levels of miRNAs in different blood components may be different and consequently reflect the miRNA variations in the cancer sites to distinctive extents [38–40]. In the current study, we only focused on serum and PBMCs samples. Although miR-21 in both serum and PBMCs was proven to be potential biomarker for GC diagnosis, the elevation of miR-21 in serum, in comparison to that in PBMCs, was more profound and also demonstrated slightly better sensitivity and specificity in AUC analysis. The subtle difference of miR-21 in serum and PBMCs as GC diagnosis biomarkers discovered in our study could imply that there is a big chance that components other than serum and PBMCs (e.g., certain subclass of blood cells) may give even better results in GC diagnosis. Further research to address this mystery is warranted.

As a novel type of tumor biomarker, miRNA, in comparison to common protein biomarkers, has some unique characteristics. One difference with great importance is that miRNAs, albeit better than other RNAs in stability, are generally much less stable than proteins [41, 42]. Therefore, unlike protein-based tests, quantification of miRNAs may require extra measures to protect samples from degradation. In general, there are two ways to cope with this problem. One is to stabilize miRNAs in the sample and the other is to shorten time required for testing. In our study, to minimize miRNA degradation, samples were immediately processed for miRNA extraction after blood collection and stored at −80°C before qRT-PCR and RNase inhibitors were added during PCR analysis. Previous studies by others have also described fast detection methods like microarray [43] and whole blood detection [44, 45] of miRNA. However, to define an optimal system for miRNA detection requires extensive research in the future [42].

In sum, based on a U6-controlled miR-21 qRT-PCR quantification method, our study indicated that circulating miR-21 (serum and PBMCs) was significantly increased in GC patients and could serve as GC diagnostic biomarkers. Moreover, the diagnostic value of miR-21 in GC was sustained from GC stages I to IV.

## 5. Conclusions

Circulating miR-21 (both in serum and in PBMCs) can serve as a good biomarker for GC and could be used in diagnosis of early (stage I) and late GC (stage IV).

## Figures and Tables

**Table 1:** Association of miR-21 expression with clinicopathological features.

Variable	*N*	Serum miR-21	*p*	PBMC miR-21	*p*
Clinical stage					
I	9	32.31 (22.88–54.12)	0.790	33.01 (22.35–53.63)	0.751
II	11	31.62 (21.14–50.12)		32.42 (21.05–51.79)	
III	10	31.43 (22.31–49.33)		31.38 (21.31–49.98)	
IV	18	30.82 (23.45–41.23)		30.19 (20.45–45.94)	
Age					
<35	8	31.77 (23.28–51.02)	0.812	30.33 (22.43–50.78)	0.892
35–50	10	29.67 (23.21–50.53)		28.66 (24.32–51.22)	
51–65	18	30.25 (24.22–52.38)		30.66 (23.99–51.59)	
>66	14	32.81 (25.88–53.62)		31.67 (22.98–54.11)	
Gender					
Male	24	29.95 (23.24–49.38)	0.844	28.66 (22.42–48.78)	0.789
Female	26	30.17 (22.66–50.19)		29.32 (23.16–50.68)	
Lymphatic metastasis					
Yes	27	29.99 (25.87–51.12)	0.678	31.54 (24.55–51.66)	0.643
No	23	30.23 (24.78–50.22)		32.11 (24.11–50.35)	
Differentiation degree					
Low	12	29.87 (24.68–49.88)	0.99	30.22 (23.33–51.66)	0.89
Middle	17	30.19 (24.98–50.86)		30.12 (23.02–51.76)	
High	21	30.21 (25.56–51.25)		31.01 (24.45–51.87)	
Surgery					
Yes	24	30.55 (24.97–50.15)	0.551	31.44 (24.29–51.54)	0.521
No	26	29.78 (23.99–51.21)		30.22 (23.12–50.18)	

**Table 2:** Association of miR-21, CEA, and CA125 expression with the clinical stage.

Clinical stage	*N*	Serum miR-21		miR-21 in PMBCs		CA199		CEA	
		Expression	Positive rate (%)	Expression	Positive rate (%)	Expression (U/mL)	Positive rate (%)	Expression (U/mL)	Positive rate (%)
I	9	32.41 (22.53–52.13)	88.88^∗#^ (8/9)	33.12 (23.55–52.32)	88.88^∗#^ (7/9)	22.66 (8.23–55.12)	44.44 (4/9)	16.3 (8.75–54.43)	33.33 (3/9)
II	11	32.33 (21.65–52.77)	90.90^∗#^ (10/11)	31.22 (22.55–50.11)	81.81^∗#^ (9/11)	30.85 (22.89–89.43)	54.54 (6/11)	48.91 (19.75–78.93)	45.45 (5/11)
III	10	33.08 (23.01–53.68)	90.00^∗#^ (9/10)	31.83 (22.79–49.94)	90.00^∗#^ (9/10)	48.44 (33.77–88.88)	40.00 (4/10)	57.99 (21.33–88.12)	50.00 (5/10)
IV	18	31.11 (19.65–49.44)	94.44^∗#^ (17/18)	30.88 (23.01–50.65)	94.44^∗#^ (17/18)	67.82 (45.75–120.93)	61.11 (11/18)	66.8 (26.57–138.64)	55.55 (10/18)
Total	**50**	**32.56 (22.24–54.17) **	88.00^∗#^ ** (44/50)**	**31.75 (22.62–48.98) **	84.00^∗#^ ** (81/90)**	**46.99 (29.53–100.11) **	**50.00 (25/50)**	**36.77 (22.11–88.65) **	**46.00 (23/50)**

^∗^ *p* < 0.05 (versus CEA).

^#^ *p* < 0.05 (versus CA125).
